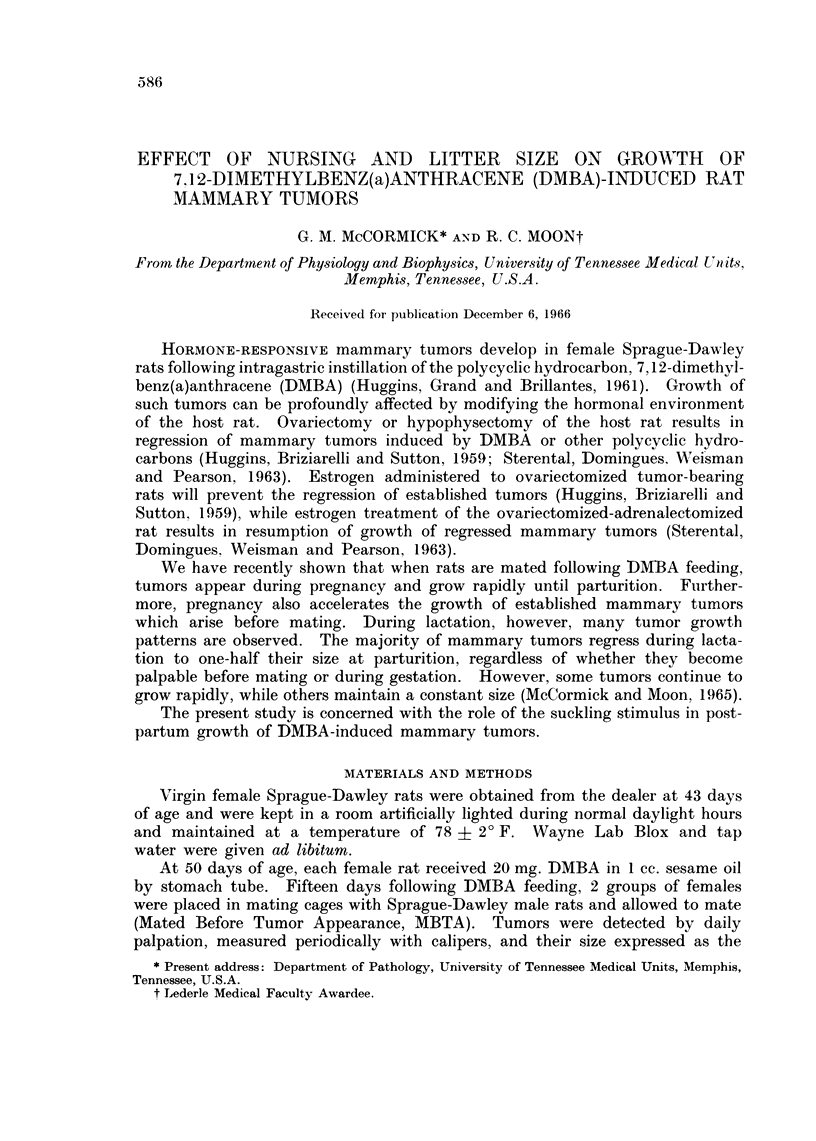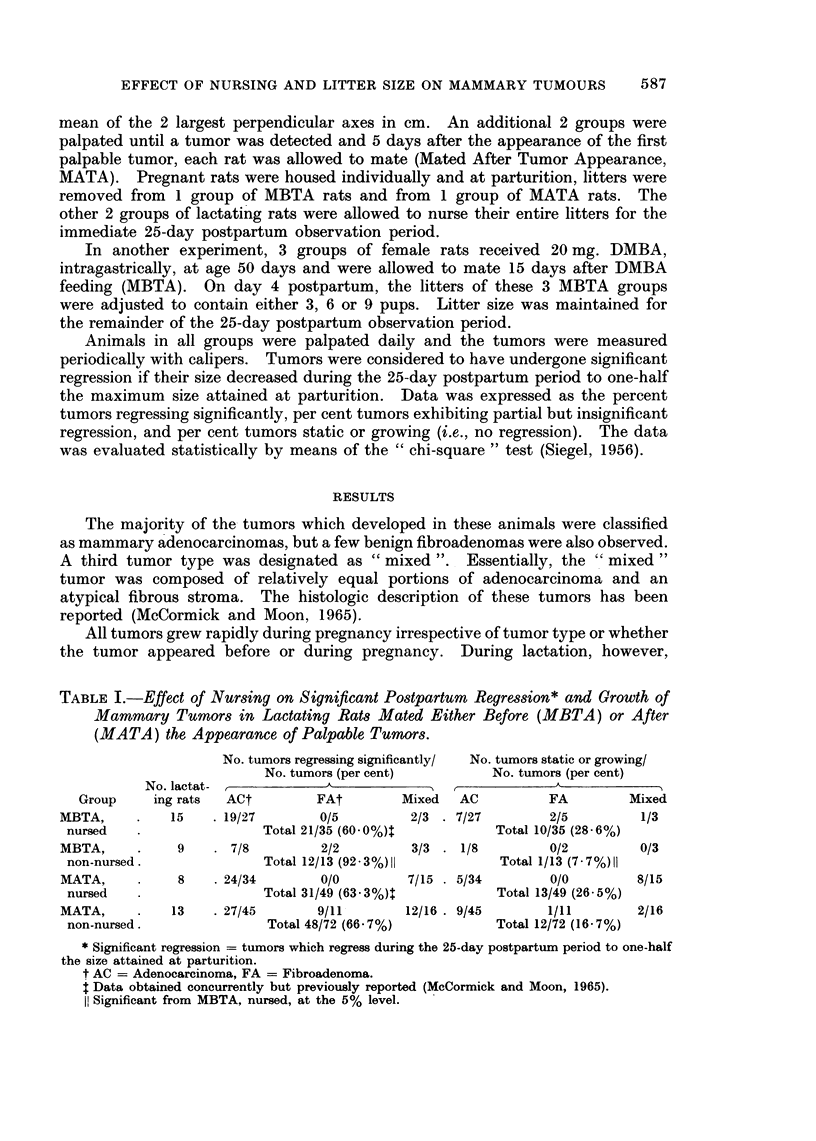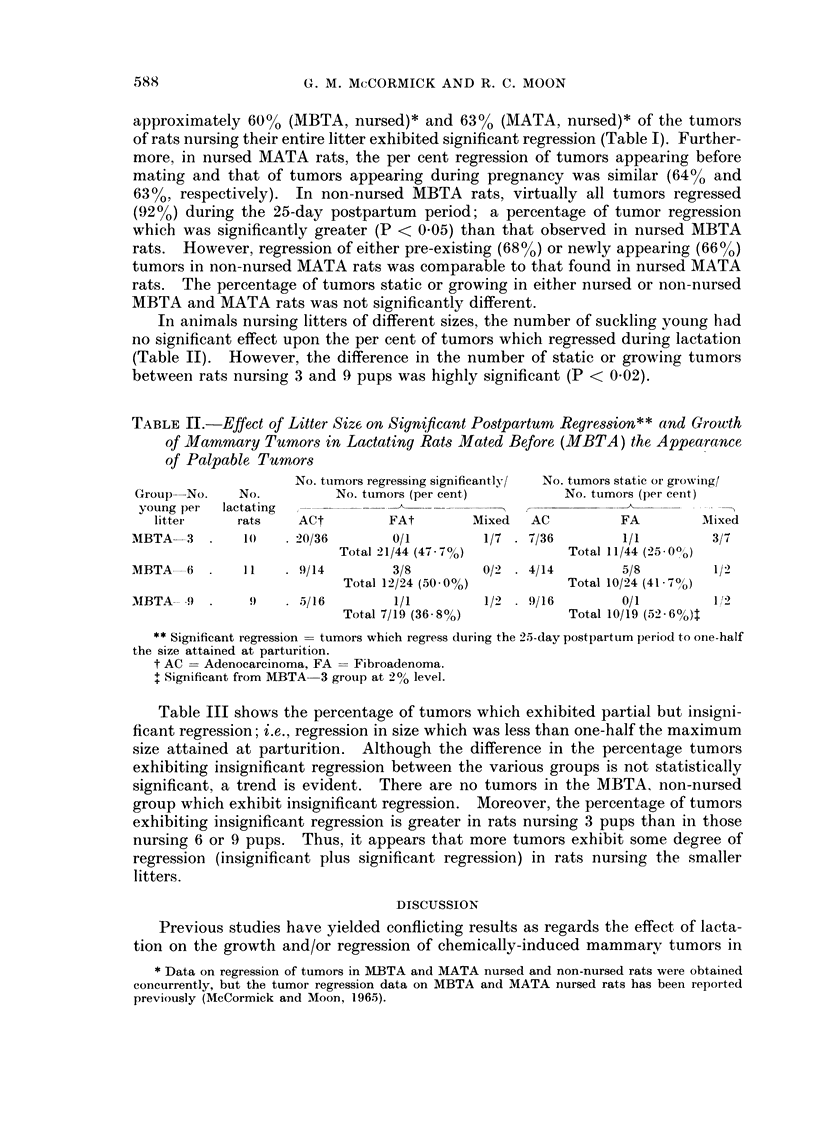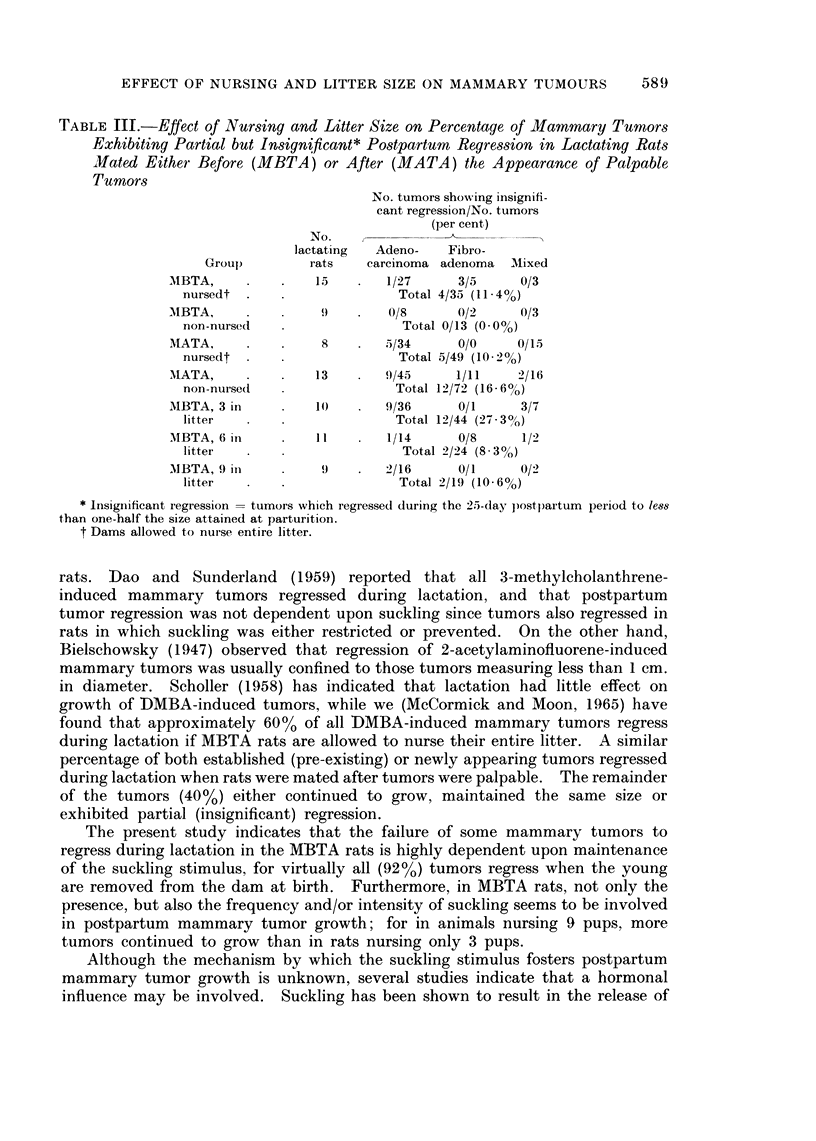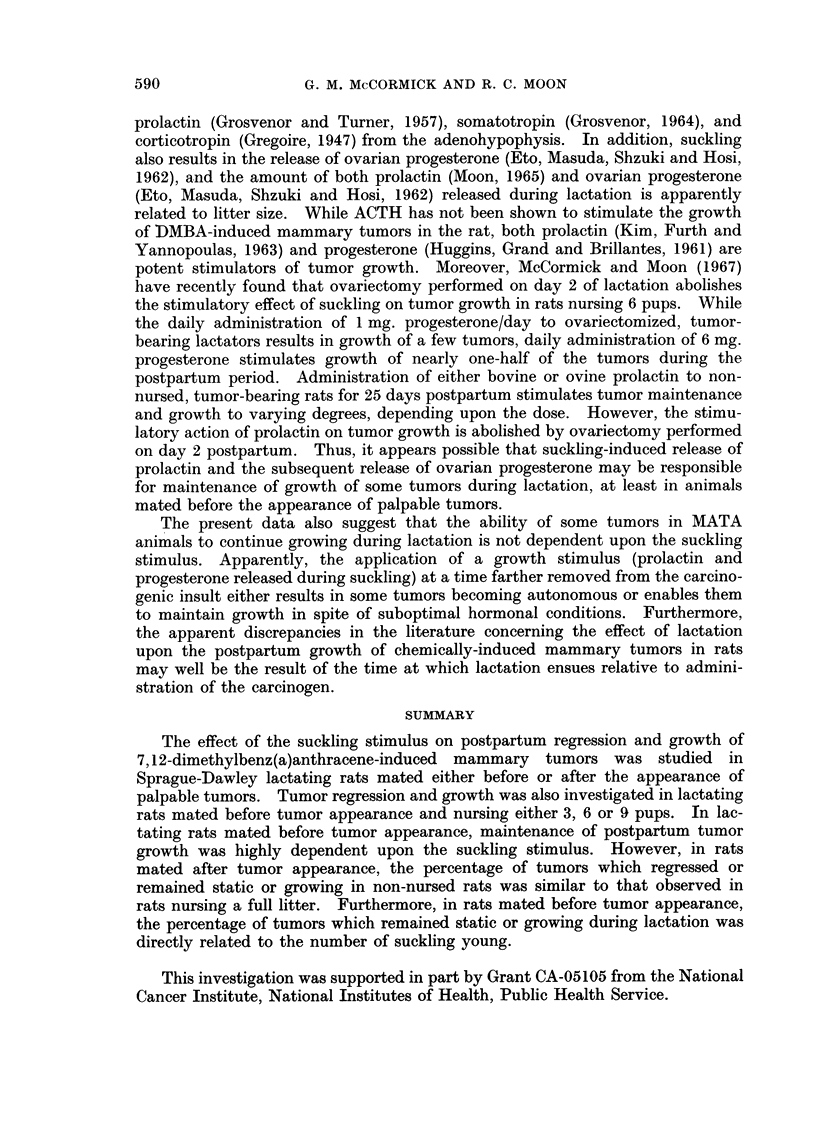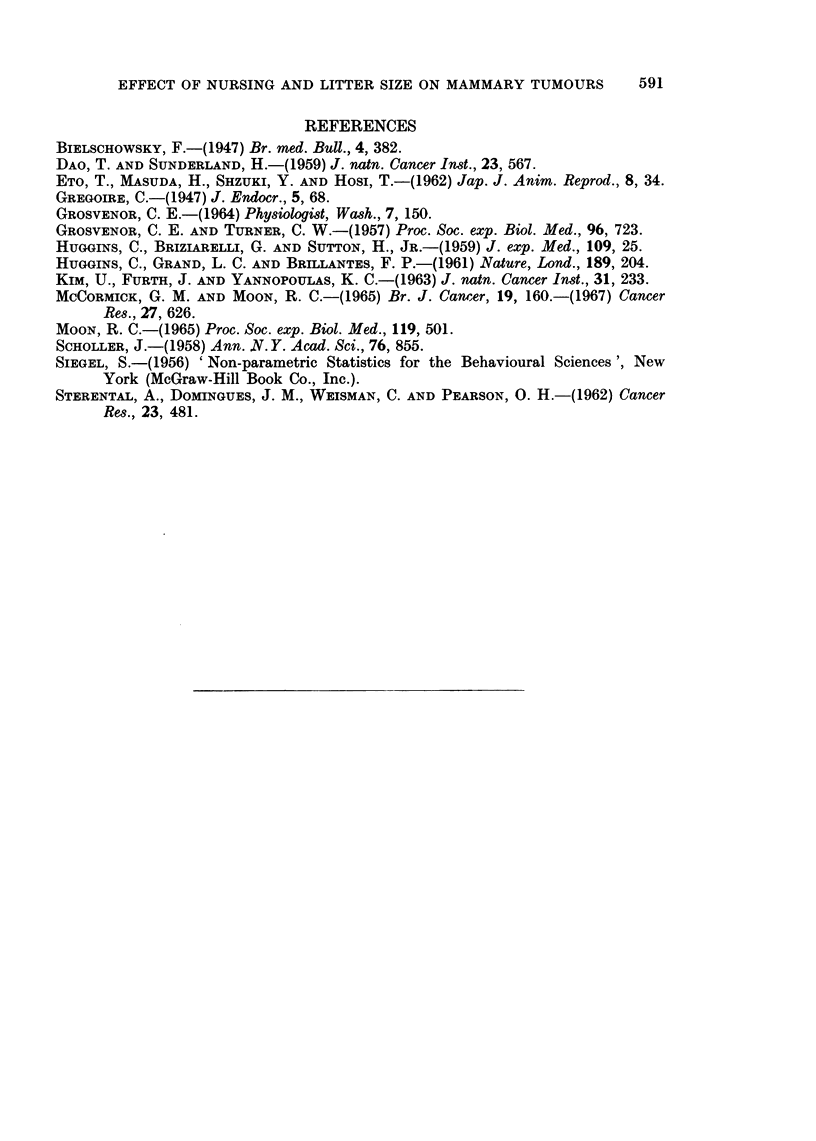# Effect of nursing and litter size on growth of 7,12-dimethylbenz(a)anthracene (DMBA)-induced rat mammary tumors.

**DOI:** 10.1038/bjc.1967.68

**Published:** 1967-09

**Authors:** G. M. McCormick, R. C. Moon


### EFFECT OF NURSING AND LITTER SIZE ON GROWTH OF 7,12-DIMETHYLBENZ(a)ANTHRACENE (DMBA)-INDUCED RAT MAMMARY TUMORS

G. M. McCORMICK* AND R. C. MOON†

*From the Department of Physiology and Biophysics, University of Tennessee Medical Units, Memphis, Tennessee, U.S.A.*

Received for publication December 6, 1966

HORMONE-RESPONSIVE mammary tumors develop in female Sprague-Dawley rats following intragastric instillation of the polycyclic hydrocarbon, 7,12-dimethylbenz(a)anthracene (DMBA) (Huggins, Grand and Brillantes, 1961). Growth of such tumors can be profoundly affected by modifying the hormonal environment of the host rat. Ovariectomy or hypophysectomy of the host rat results in regression of mammary tumors induced by DMBA or other polycyclic hydrocarbons (Huggins, Briziarelli and Sutton, 1959; Sterental, Domingues, Weisman and Pearson, 1963). Estrogen administered to ovariectomized tumor-bearing rats will prevent the regression of established tumors (Huggins, Briziarelli and Sutton, 1959), while estrogen treatment of the ovariectomized-adrenalectomized rat results in resumption of growth of regressed mammary tumors (Sterental, Domingues, Weisman and Pearson, 1963).

We have recently shown that when rats are mated following DMBA feeding, tumors appear during pregnancy and grow rapidly until parturition. Furthermore, pregnancy also accelerates the growth of established mammary tumors which arise before mating. During lactation, however, many tumor growth patterns are observed. The majority of mammary tumors regress during lactation to one-half their size at parturition, regardless of whether they become palpable before mating or during gestation. However, some tumors continue to grow rapidly, while others maintain a constant size (McCormick and Moon, 1965).

The present study is concerned with the role of the suckling stimulus in postpartum growth of DMBA-induced mammary tumors.

### MATERIALS AND METHODS

Virgin female Sprague-Dawley rats were obtained from the dealer at 43 days of age and were kept in a room artificially lighted during normal daylight hours and maintained at a temperature of $78 \pm 2^{\circ}$ F. Wayne Lab Blox and tap water were given *ad libitum.*

At 50 days of age, each female rat received 20 mg. DMBA in 1 cc. sesame oil by stomach tube. Fifteen days following DMBA feeding, 2 groups of females were placed in mating cages with Sprague-Dawley male rats and allowed to mate (Mated Before Tumor Appearance, MBTA). Tumors were detected by daily palpation, measured periodically with calipers, and their size expressed as the

* Present address: Department of Pathology, University of Tennessee Medical Units, Memphis, Tennessee, U.S.A.

† Lederle Medical Faculty Awardee.

mean of the 2 largest perpendicular axes in cm. An additional 2 groups were palpated until a tumor was detected and 5 days after the appearance of the first palpable tumor, each rat was allowed to mate (Mated After Tumor Appearance, MATA). Pregnant rats were housed individually and at parturition, litters were removed from 1 group of MBTA rats and from 1 group of MATA rats. The other 2 groups of lactating rats were allowed to nurse their entire litters for the immediate 25-day postpartum observation period.

In another experiment, 3 groups of female rats received 20 mg. DMBA, intragastrically, at age 50 days and were allowed to mate 15 days after DMBA feeding (MBTA). On day 4 postpartum, the litters of these 3 MBTA groups were adjusted to contain either 3, 6 or 9 pups. Litter size was maintained for the remainder of the 25-day postpartum observation period.

Animals in all groups were palpated daily and the tumors were measured periodically with calipers. Tumors were considered to have undergone significant regression if their size decreased during the 25-day postpartum period to one-half the maximum size attained at parturition. Data was expressed as the percent tumors regressing significantly, per cent tumors exhibiting partial but insignificant regression, and per cent tumors static or growing (*i.e.*, no regression). The data was evaluated statistically by means of the " chi-square " test (Siegel, 1956).

### RESULTS

The majority of the tumors which developed in these animals were classified as mammary adenocarcinomas, but a few benign fibroadenomas were also observed. A third tumor type was designated as " mixed ". Essentially, the " mixed " tumor was composed of relatively equal portions of adenocarcinoma and an atypical fibrous stroma. The histologic description of these tumors has been reported (McCormick and Moon, 1965).

All tumors grew rapidly during pregnancy irrespective of tumor type or whether the tumor appeared before or during pregnancy. During lactation, however,

TABLE I.—*Effect of Nursing on Significant Postpartum Regression* and Growth of Mammary Tumors in Lactating Rats Mated Either Before (MBTA) or After (MATA) the Appearance of Palpable Tumors.*

| | | No. tumors regressing significantly/ No. tumors (per cent) | | | No. tumors static or growing/ No. tumors (per cent) | | |
|---|---|---|---|---|---|---|---|
| Group | No. lactating rats | AC† | FA† | Mixed | AC | FA | Mixed |
| MBTA, nursed | 15 | 19/27 | 0/5 | 2/3 | 7/27 | 2/5 | 1/3 |
| | | | Total 21/35 (60·0%)‡ | | | Total 10/35 (28·6%) | |
| MBTA, non-nursed | 9 | 7/8 | 2/2 | 3/3 | 1/8 | 0/2 | 0/3 |
| | | | Total 12/13 (92·3%)‖ | | | Total 1/13 (7·7%)‖ | |
| MATA, nursed | 8 | 24/34 | 0/0 | 7/15 | 5/34 | 0/0 | 8/15 |
| | | | Total 31/49 (63·3%)‡ | | | Total 13/49 (26·5%) | |
| MATA, non-nursed | 13 | 27/45 | 9/11 | 12/16 | 9/45 | 1/11 | 2/16 |
| | | | Total 48/72 (66·7%) | | | Total 12/72 (16·7%) | |

* Significant regression = tumors which regress during the 25-day postpartum period to one-half the size attained at parturition.

† AC = Adenocarcinoma, FA = Fibroadenoma.

‡ Data obtained concurrently but previously reported (McCormick and Moon, 1965).

‖ Significant from MBTA, nursed, at the 5% level.

approximately 60% (MBTA, nursed)* and 63% (MATA, nursed)* of the tumors of rats nursing their entire litter exhibited significant regression (Table I). Furthermore, in nursed MATA rats, the per cent regression of tumors appearing before mating and that of tumors appearing during pregnancy was similar (64% and 63%, respectively). In non-nursed MBTA rats, virtually all tumors regressed (92%) during the 25-day postpartum period; a percentage of tumor regression which was significantly greater ($P < 0{\cdot}05$) than that observed in nursed MBTA rats. However, regression of either pre-existing (68%) or newly appearing (66%) tumors in non-nursed MATA rats was comparable to that found in nursed MATA rats. The percentage of tumors static or growing in either nursed or non-nursed MBTA and MATA rats was not significantly different.

In animals nursing litters of different sizes, the number of suckling young had no significant effect upon the per cent of tumors which regressed during lactation (Table II). However, the difference in the number of static or growing tumors between rats nursing 3 and 9 pups was highly significant ($P < 0{\cdot}02$).

TABLE II.—*Effect of Litter Size on Significant Postpartum Regression*** *and Growth of Mammary Tumors in Lactating Rats Mated Before (MBTA) the Appearance of Palpable Tumors*

| Group—No. young per litter | No. lactating rats | No. tumors regressing significantly/ No. tumors (per cent) | | | No. tumors static or growing/ No. tumors (per cent) | | |
|---|---|---|---|---|---|---|---|
| | | AC† | FA† | Mixed | AC | FA | Mixed |
| MBTA—3 | 10 | 20/36 | 0/1 | 1/7 | 7/36 | 1/1 | 3/7 |
| | | | Total 21/44 (47·7%) | | | Total 11/44 (25·0%) | |
| MBTA—6 | 11 | 9/14 | 3/8 | 0/2 | 4/14 | 5/8 | 1/2 |
| | | | Total 12/24 (50·0%) | | | Total 10/24 (41·7%) | |
| MBTA—9 | 9 | 5/16 | 1/1 | 1/2 | 9/16 | 0/1 | 1/2 |
| | | | Total 7/19 (36·8%) | | | Total 10/19 (52·6%)‡ | |

** Significant regression = tumors which regress during the 25-day postpartum period to one-half the size attained at parturition.
† AC = Adenocarcinoma, FA = Fibroadenoma.
‡ Significant from MBTA—3 group at 2% level.

Table III shows the percentage of tumors which exhibited partial but insignificant regression; *i.e.*, regression in size which was less than one-half the maximum size attained at parturition. Although the difference in the percentage tumors exhibiting insignificant regression between the various groups is not statistically significant, a trend is evident. There are no tumors in the MBTA, non-nursed group which exhibit insignificant regression. Moreover, the percentage of tumors exhibiting insignificant regression is greater in rats nursing 3 pups than in those nursing 6 or 9 pups. Thus, it appears that more tumors exhibit some degree of regression (insignificant plus significant regression) in rats nursing the smaller litters.

### DISCUSSION

Previous studies have yielded conflicting results as regards the effect of lactation on the growth and/or regression of chemically-induced mammary tumors in

* Data on regression of tumors in MBTA and MATA nursed and non-nursed rats were obtained concurrently, but the tumor regression data on MBTA and MATA nursed rats has been reported previously (McCormick and Moon, 1965).

TABLE III.—*Effect of Nursing and Litter Size on Percentage of Mammary Tumors Exhibiting Partial but Insignificant* Postpartum Regression in Lactating Rats Mated Either Before (MBTA) or After (MATA) the Appearance of Palpable Tumors*

| Group | No. lactating rats | No. tumors showing insignificant regression/No. tumors (per cent) Adeno-carcinoma | Fibro-adenoma | Mixed |
|---|---|---|---|---|
| MBTA, nursed† | 15 | 1/27 | 3/5 | 0/3 |
| | | Total 4/35 (11·4%) | | |
| MBTA, non-nursed | 9 | 0/8 | 0/2 | 0/3 |
| | | Total 0/13 (0·0%) | | |
| MATA, nursed† | 8 | 5/34 | 0/0 | 0/15 |
| | | Total 5/49 (10·2%) | | |
| MATA, non-nursed | 13 | 9/45 | 1/11 | 2/16 |
| | | Total 12/72 (16·6%) | | |
| MBTA, 3 in litter | 10 | 9/36 | 0/1 | 3/7 |
| | | Total 12/44 (27·3%) | | |
| MBTA, 6 in litter | 11 | 1/14 | 0/8 | 1/2 |
| | | Total 2/24 (8·3%) | | |
| MBTA, 9 in litter | 9 | 2/16 | 0/1 | 0/2 |
| | | Total 2/19 (10·6%) | | |

* Insignificant regression = tumors which regressed during the 25-day postpartum period to *less* than one-half the size attained at parturition.

† Dams allowed to nurse entire litter.

rats. Dao and Sunderland (1959) reported that all 3-methylcholanthrene-induced mammary tumors regressed during lactation, and that postpartum tumor regression was not dependent upon suckling since tumors also regressed in rats in which suckling was either restricted or prevented. On the other hand, Bielschowsky (1947) observed that regression of 2-acetylaminofluorene-induced mammary tumors was usually confined to those tumors measuring less than 1 cm. in diameter. Scholler (1958) has indicated that lactation had little effect on growth of DMBA-induced tumors, while we (McCormick and Moon, 1965) have found that approximately 60% of all DMBA-induced mammary tumors regress during lactation if MBTA rats are allowed to nurse their entire litter. A similar percentage of both established (pre-existing) or newly appearing tumors regressed during lactation when rats were mated after tumors were palpable. The remainder of the tumors (40%) either continued to grow, maintained the same size or exhibited partial (insignificant) regression.

The present study indicates that the failure of some mammary tumors to regress during lactation in the MBTA rats is highly dependent upon maintenance of the suckling stimulus, for virtually all (92%) tumors regress when the young are removed from the dam at birth. Furthermore, in MBTA rats, not only the presence, but also the frequency and/or intensity of suckling seems to be involved in postpartum mammary tumor growth; for in animals nursing 9 pups, more tumors continued to grow than in rats nursing only 3 pups.

Although the mechanism by which the suckling stimulus fosters postpartum mammary tumor growth is unknown, several studies indicate that a hormonal influence may be involved. Suckling has been shown to result in the release of prolactin (Grosvenor and Turner, 1957), somatotropin (Grosvenor, 1964), and corticotropin (Gregoire, 1947) from the adenohypophysis. In addition, suckling also results in the release of ovarian progesterone (Eto, Masuda, Shzuki and Hosi, 1962), and the amount of both prolactin (Moon, 1965) and ovarian progesterone (Eto, Masuda, Shzuki and Hosi, 1962) released during lactation is apparently related to litter size. While ACTH has not been shown to stimulate the growth of DMBA-induced mammary tumors in the rat, both prolactin (Kim, Furth and Yannopoulas, 1963) and progesterone (Huggins, Grand and Brillantes, 1961) are potent stimulators of tumor growth. Moreover, McCormick and Moon (1967) have recently found that ovariectomy performed on day 2 of lactation abolishes the stimulatory effect of suckling on tumor growth in rats nursing 6 pups. While the daily administration of 1 mg. progesterone/day to ovariectomized, tumor-bearing lactators results in growth of a few tumors, daily administration of 6 mg. progesterone stimulates growth of nearly one-half of the tumors during the postpartum period. Administration of either bovine or ovine prolactin to non-nursed, tumor-bearing rats for 25 days postpartum stimulates tumor maintenance and growth to varying degrees, depending upon the dose. However, the stimulatory action of prolactin on tumor growth is abolished by ovariectomy performed on day 2 postpartum. Thus, it appears possible that suckling-induced release of prolactin and the subsequent release of ovarian progesterone may be responsible for maintenance of growth of some tumors during lactation, at least in animals mated before the appearance of palpable tumors.

The present data also suggest that the ability of some tumors in MATA animals to continue growing during lactation is not dependent upon the suckling stimulus. Apparently, the application of a growth stimulus (prolactin and progesterone released during suckling) at a time farther removed from the carcinogenic insult either results in some tumors becoming autonomous or enables them to maintain growth in spite of suboptimal hormonal conditions. Furthermore, the apparent discrepancies in the literature concerning the effect of lactation upon the postpartum growth of chemically-induced mammary tumors in rats may well be the result of the time at which lactation ensues relative to administration of the carcinogen.

### SUMMARY

The effect of the suckling stimulus on postpartum regression and growth of 7,12-dimethylbenz(a)anthracene-induced mammary tumors was studied in Sprague-Dawley lactating rats mated either before or after the appearance of palpable tumors. Tumor regression and growth was also investigated in lactating rats mated before tumor appearance and nursing either 3, 6 or 9 pups. In lactating rats mated before tumor appearance, maintenance of postpartum tumor growth was highly dependent upon the suckling stimulus. However, in rats mated after tumor appearance, the percentage of tumors which regressed or remained static or growing in non-nursed rats was similar to that observed in rats nursing a full litter. Furthermore, in rats mated before tumor appearance, the percentage of tumors which remained static or growing during lactation was directly related to the number of suckling young.

This investigation was supported in part by Grant CA-05105 from the National Cancer Institute, National Institutes of Health, Public Health Service.

### REFERENCES

BIELSCHOWSKY, F.—(1947) *Br. med. Bull.*, **4**, 382.

DAO, T. AND SUNDERLAND, H.—(1959) *J. natn. Cancer Inst.*, **23**, 567.

ETO, T., MASUDA, H., SHZUKI, Y. AND HOSI, T.—(1962) *Jap. J. Anim. Reprod.*, **8**, 34.

GREGOIRE, C.—(1947) *J. Endocr.*, **5**, 68.

GROSVENOR, C. E.—(1964) *Physiologist, Wash.*, **7**, 150.

GROSVENOR, C. E. AND TURNER, C. W.—(1957) *Proc. Soc. exp. Biol. Med.*, **96**, 723.

HUGGINS, C., BRIZIARELLI, G. AND SUTTON, H., JR.—(1959) *J. exp. Med.*, **109**, 25.

HUGGINS, C., GRAND, L. C. AND BRILLANTES, F. P.—(1961) *Nature, Lond.*, **189**, 204.

KIM, U., FURTH, J. AND YANNOPOULAS, K. C.—(1963) *J. natn. Cancer Inst.*, **31**, 233.

MCCORMICK, G. M. AND MOON, R. C.—(1965) *Br. J. Cancer*, **19**, 160.—(1967) *Cancer Res.*, **27**, 626.

MOON, R. C.—(1965) *Proc. Soc. exp. Biol. Med.*, **119**, 501.

SCHOLLER, J.—(1958) *Ann. N.Y. Acad. Sci.*, **76**, 855.

SIEGEL, S.—(1956) 'Non-parametric Statistics for the Behavioural Sciences', New York (McGraw-Hill Book Co., Inc.).

STERENTAL, A., DOMINGUES, J. M., WEISMAN, C. AND PEARSON, O. H.—(1962) *Cancer Res.*, **23**, 481.